# Barley Leaves Improves Loperamide-Induced Constipation via Gut Barrier and Microbiota Modulation in Mice

**DOI:** 10.3390/foods15010095

**Published:** 2025-12-29

**Authors:** Yuting Xu, Zhiqian Wu, Matthew Lee Cohoon, Mengting Ma, Zhongquan Sui, Harold Corke

**Affiliations:** 1 Department of Food Science & Technology, School of Agriculture and Biology, Shanghai Jiao Tong University, Shanghai 200240, China; nov16xyt@sjtu.edu.cn (Y.X.);; 2 Department of Biotechnology and Food Engineering, Guangdong Technion-Israel Institute of Technology, Shantou 515063, China; 3 Faculty of Biotechnology and Food Engineering, Technion-Israel Institute of Technology, Haifa 3200003, Israel

**Keywords:** barley leaves, constipation, serum neurotransmitters, short chain fatty acids, gut microbiota regulation

## Abstract

Constipation is a common gastrointestinal disorder that seriously affects quality of life and is associated with multiple secondary complications. Barley leaves (BLs) have been suggested as potential functional foods for constipation prevention. Here, we investigated the preventive effects of common barley leaves (CBLs) and hulless barley leaves (HBLs) in a loperamide-induced constipation model in C57BL/6 mice. Both BLs improved stool parameters and gastrointestinal transit. Notably, high-dose HBLs increased stool weight to 263.84 ± 66.70 mg and stool amount to 250.20 ± 66.88 pellets, which were 12.7 and 11.1 times higher than those in the model group, respectively. BLs also modulated gut motility-related hormones (MTL, SP, Gas, SS, and VIP) and normalized colonic *AQP3*, *AQP4*, and *5-HT*_4_*R* expression levels. Furthermore, BLs enhanced SCFAs production and modulated gut microbiota by increasing *Bacteroides* abundance and decreasing *Akkermansia* abundance. CBLs and HBLs also exhibited distinct mechanisms. High-dose CBLs affected *SERT* expression, whereas HBLs uniquely decreased *Alistipes* abundance and increased SCFA production. These findings suggest that BLs may help prevent loperamide-induced constipation in mice by modulating the gut barrier and microbiota. Future studies should identify key active components and validate efficacy in longer-term and clinical studies.

## 1. Introduction

Constipation is a widespread bowel disorder that influences approximately 12–19% of the global population and significantly impacts daily life and productivity, leading to considerable personal and societal burdens [1]. It is clinically characterized by various uncomfortable symptoms, including difficult defecation, infrequent bowel movements, or a sensation of incomplete evacuation [2]. Long-term constipation can lead to serious gastrointestinal diseases, such as accumulation of intestinal toxins, irritable bowel syndrome, and colon cancer [3], and is proposed to be associated with cardiovascular, respiratory, and nervous systems [4]. Thus, there is an urgent need to identify safe and effective strategies for the prevention and treatment of constipation. Current pharmacological interventions primarily include laxatives, secretagogues, serotonergic agonists, and suppositories, all of which are associated with significant side effects such as flatulence, bloating, and abdominal pain [5]. Therefore, plant-derived functional foods with minimal adverse effects have attracted increasing interest as alternative approaches for preventing and alleviating constipation, thereby potentially reducing the incidence of related diseases.

Barley (*Hordeum vulgare* L.) is a highly significant crop in global agriculture and can be divided into two primary types based on grain characteristics: hulled barley and hulless (naked) barley, with hulless barley (*Hordeum vulgare* L. var. *nudum* Hook.f.) as a typical example of the latter [6]. Barley leaves (BLs) are derived from the young grass of barley, the primary ingredient in a popular green functional drink in Asian countries [7]. Common barley leaves (CBLs), which are derived from hulled barley (i.e., hulled barley leaves), are rich in nutrients such as dietary fiber, protein, minerals, vitamins, and flavonoids [7]. In contrast, hulless barley leaves (HBLs) have been reported to contain higher contents of protein, amylose, and fiber and a lower lipid content than CBLs, attributed to their unique high-altitude growing environment, distinct climatic conditions, and genetics background [8]. Notably, these compositional traits can vary among barley varieties and growing environments [6]. These compositional differences suggest that CBLs and HBLs may exert distinct physiological effects.

Accumulating evidence indicates that BLs can prevent obesity, depression, and gastrointestinal disorders [9,10,11,12,13]. Polysaccharides from HBLs exhibit strong anti-proliferative activity against HT29, Caco-2, 4T1, and CT26 cancer cells [10]. Yan et al. reported that CBL polysaccharides can alleviate hyperlipidemia by regulating dyslipidemia, relieving liver injury, and reshaping intestinal flora structure [11]. Moreover, CBLs have been shown to influence gastrointestinal function in healthy human volunteers and patients with mild constipation [12], and studies in rats have demonstrated that water-insoluble dietary fiber from CBLs can increased fecal weight and shorten gastrointestinal transit time by promoting probiotic growth [13]. Nevertheless, previous studies on BLs have primarily focused on general nutritional and health benefits or on CBLs alone, with limited attention paid to their specific role and mechanisms in preventing constipation. It remains unclear how BLs modulate intestinal barrier function, neurotransmitter signaling, gut microbiota, and short-chain fatty acid (SCFA) production in the context of constipation and whether the distinct nutritional profiles of CBLs and HBLs translate into different protective outcomes.

Therefore, this study aimed to evaluate the preventive effects of CBL and HBL interventions on loperamide-induced constipation in C57BL/6 mice. We assessed fecal parameters, gastrointestinal transit efficiency, colonic histomorphology, gene expression related to intestinal barrier function, serum neurotransmitter levels, gut microbiota structure, and short-chain fatty acids (SCFAs). By examining CBLs and HBLs within the same experimental framework, we sought to clarify the mechanisms underlying BL-mediated protection against constipation and to determine the scientific relevance of using different barley leaf types as potential ingredients in functional foods aimed at preventing constipation.

## 2. Material and Methods

### 2.1. Materials

CBLs and HBLs, sourced from Shanghai Ganya Biotechnology Co., Ltd. (Shanghai, China), underwent cleaning, freeze-drying, grinding into a fine green powder, and sieving using a 40-mesh screen. Loperamide hydrochloride was commercially acquired from Xi’an Janssen Pharmaceutical Co., Ltd. (Xi’an, China). Naloxone hydrochloride was obtained from Jilin Zhenghe Pharmaceutical Group Co., Ltd. (Tonghua, China). Arabic gum and methyl tertiary butyl ether were commercially acquired from Sinopharm Chemical Reagent Co., Ltd. (Shanghai, China). Activated carbon, sulfuric acid, and 2-methylvaleric acid were obtained from Aradin Bio-chem Technology Co., Ltd. (Shanghai, China).

### 2.2. Compositional Analysis

#### 2.2.1. Determination of Total Phenols and Total Flavonoids

The dried powder (0.5 g) was combined with 10 mL of 40% (*v*/*v*) methanol and maintained at 45 °C for 1.5 h. Once cooled, the mixture underwent centrifugation at 4000× *g* for 10 min, and the resulting supernatant was collected. The total phenolics and flavonoids content of CBLs and HBLs was determined as described previously [14]. The measurements were carried out in triplicate.

#### 2.2.2. Protein Content

The protein of CLBs and HLBs was estimated by using a standard Kjeldahl method (*N* % × 6.25), as previously described [15]. The measurements were carried out in triplicate.

#### 2.2.3. Dietary Fiber Content

The dietary fiber content of CLBs and HLBs was measured using an enzymatic–gravimetric procedure (AOAC Method 991.43) [16]. The measurements were carried out in triplicate.

### 2.3. Animal Experiments

All animal procedures were carried out under the authorization of the Animal Ethics Committee of Shanghai Jiao Tong University (No. A2023227-1). A total of 84 male C57BL/6 mice (7–8 weeks, 18–22 g) were randomly assigned to two cohorts to perform a defecation test and a gastrointestinal transit test, respectively. After seven days of adaptive feeding, mice in each cohort were further randomized into seven groups (*n* = 6 per group): normal control group (NC, saline solution), model group (MC, saline solution), positive drug group (PC, saline solution), low-dose CLBs powder group (L-CB, 0.1 g/kg bw), high-dose CLBs powder group (H-CB, 0.5 g/kg bw), low-dose HLBs powder group (L-HB, 0.1 g/kg bw), and high-dose HLBs powder group (H-LB, 0.5 g/kg bw). The doses of CBLs and HBLs (0.1 and 0.5 g/kg bw) were selected based on our preliminary dose-ranging experiment to ensure good tolerability and to allow evaluation of potential dose-dependent preventive effects during the 7-day intervention. Saline solution, CLBs powder, and HLBs powder were administered by gavage for seven days.

### 2.4. Defecation Test

Gum arabic (100 g) was dissolved in 1000 mL of deionized water and heated to clarity. Afterward, 50 g of activated carbon solution was added, and the solution was reheated three times.

**Figure 1:**
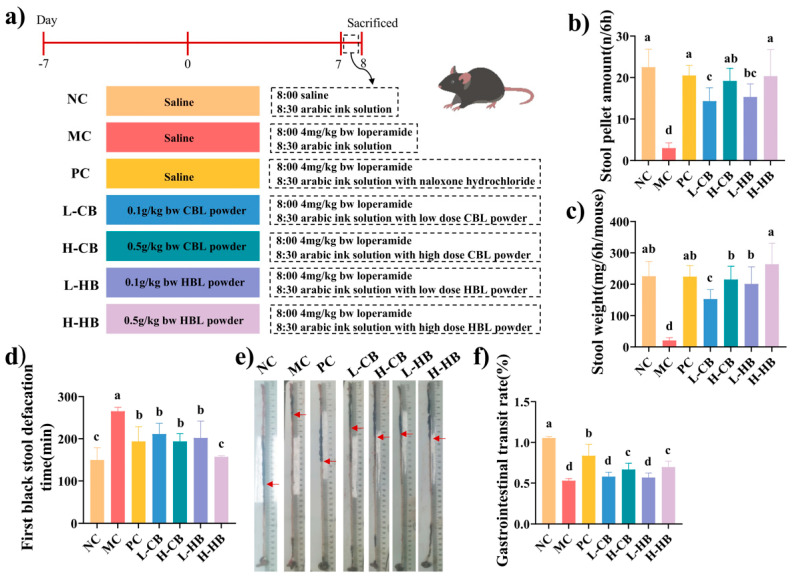
The effects of CBLs and HBLs on gastrointestinal indicators in constipated mice. (**a**) The animal experimental scheme, (**b**) stool pellet amount, (**c**) stool weight, (**d**) first black stool defecation, and (**e**) gastrointestinal transport rate experimental picture. (**f**) Gastrointestinal transit rate. The red arrows indicate the ink front position in the small intestine for transit-rate calculation. Results were expressed as mean ± SD (*n* = 6). Different small letters indicate that values are significantly different at the *p* < 0.05 level.

As shown in Figure 1a, after seven days of intervention, the mice were treated with loperamide hydrochloride (4 mg/kg bw) or normal saline (NC group). Thirty minutes later, arabic ink solution was intragastrical administered to the NC and MC groups, while the remaining groups (L-CB, H-CB, L-HB, H-HB) received the ink solution supplemented with their respective intervention powders. Naloxone hydrochloride was selected as a positive drug. Mice in the PC group were administered the arabic ink solution with naloxone hydrochloride (10 mg/kg bw) by gavage. Over a 6 h span, feces were harvested, and the amount and weight of fecal pellets were noted. The time to the first black stool, indicating the interval from activated carbon feeding to initial black feces excretion, was also evaluated.

### 2.5. Gastrointestinal Transit

The mice were given loperamide hydrochloride and different arabic inks, similar to the procedure in Section 2.4. After 25 min, the animals were sacrificed and underwent dissection to obtain the entire segment of the small intestine, extending from the pylorus to the ileocecal valve. This measurement was taken as the overall length of the small intestine. The extent of ink migration and the total intestinal length were then recorded. Intestinal motility was expressed as the ratio of ink advancement to the full length of the small intestine, calculated using the following Formula (1).

(1) ink propulsion rate (%) = ink propulsive length/total intestinal length × 100%

Then, the small intestine propulsion rate was derived from Formula (2).

(2) $$small\;intestine\;propulsion\;rate=Sin^{-1}\sqrt{ink\;propulsion\;rate}\;$$

### 2.6. Gene Expression in the Colon Tissues

The clone tissues were isolated and preserved at −80 °C. The total RNA was extracted and transcribed into cDNA according to the instructions of PrimeScript^TM^ RT Reagent Kit (Takara, Kusatsu, Shiga, Japan). Primers were synthesized by Sangon Bioengineering Co., Ltd. (Shanghai, China). GAPDH served as the internal reference, and its stability was verified across all groups prior to normalization. The primer sequences are shown in Table 1.

### 2.7. Histopathological Analysis

Colonic samples from three randomly selected mice per group were preserved in 4% paraformaldehyde for at least 48 h. Following this, the tissues were infiltrated with paraffin and cut into 5 μm sections. These sections were subsequently stained using hematoxylin and eosin (H&E) and examined under a Nikon DS-U3 microscope (Tokyo, Japan).

### 2.8. Determination of Serum SS, VIP, SP, MTL and Gas Levels

SS, VIP, SP, MTL, and Gas were measured (*n* = 6 per group) using commercial ELISA kits (Shanghai Yuanju Bio-Technology Venter, Shanghai, China).

### 2.9. Gut Microbiota Analysis

For microbiota analysis, each group contained six mice. One fecal pellet was collected from each mouse and combined to generate one pooled sample (six pellets per pooled sample). Three pooled fecal samples were prepared per group, and 16S rRNA sequencing was performed (*n* = 3 pooled samples per group). The gut microbiota analysis was determined as described previously [11].

### 2.10. Determination of SCFAs in Feces

The analysis of SCFAs was carried out using a previously reported protocol with slight modifications [17]. Fecal samples (*n* = 3 pooled samples per group) were processed as follows: stool (0.5 g) was added to 800 μL DI water, homogenized, and centrifuged at 13,200× *g* for 20 min. Acidification was achieved by mixing 0.4 mL of the supernatant with 100 μL of 50% sulfuric acid, after which 0.5 mL of internal standard solution was added (methyl tert-butyl ether containing 0.5 μg/mL 2-methylvalerate). Then, stool was centrifuged again under the same conditions (13,200× *g*, 20 min). The resulting supernatant was analyzed using a GC-MS system (Agilent 7890B-7000D, Palo Alto, CA, USA). Quantification was performed via an external standard calibration curve. Fecal samples were lyophilized (freeze-dried) to constant weight, and SCFA concentrations were reported as μg/g dry feces.

### 2.11. Statistical Analysis

The results were statistically analyzed using SPSS software (Version 20.0; SPSS Software, Chicago, IL, USA). Differences among groups were assessed by one-way analysis of variance (ANOVA), followed by Duncan’s post hoc test. Data are presented as mean ± standard deviation (SD). *p*-values < 0.05 were considered statistically significant. The Figures were created using Origin 2025b software (OriginLab Corporation, North Hampton, MA, USA) and GraphPad Prism version 8 (GraphPad Software, San Diego, CA, USA).

## 3. Results

### 3.1. Analysis of the Chemical Composition of CBLs and HBLs

As displayed in Table 2, both CBLs and HBLs were rich in dietary fiber and protein, with concentrations notably higher than those of common cereal grains, as previously reported [18]. HBLs contained higher contents of dietary fiber (475.7 ± 1.7 mg/g vs. 448.4 ± 2.1 mg/g) and protein (282.4 ± 1.1 mg/g vs. 219.0 ± 3.4 mg/g) than CBLs, which was also consistent with earlier findings on hulless barley leaves [8]. CBLs, however, exhibited a higher total phenolic content (21.43 ± 0.21 mg/g) than HBLs (7.42 ± 0.1 mg/g). The most pronounced difference was observed in total flavonoids content, with 20.7 ± 0.46 mg/g in CBLs, over 20 times higher than the 1.01 ± 0.06 mg/g detected in HLBs. This pattern contrasts with a previous report indicating higher total flavonoids content in hulless barley leaves [19], suggesting that flavonoid accumulation was strongly influenced by barley variety, growing conditions, harvest stage, extraction, and analytical methods [8,19]. Overall, these results confirm that BLs provide beneficial nutrients and that their phenolic and flavonoid profiles were variety-dependent, which might contribute to their differential effects on constipation prevention.

### 3.2. Effects of CBLs and HBLs on Defecation Function and Gastrointestinal Transit

Measures of four gastrointestinal indicators are shown in Figure 1b–f. In the MC group, stool weight and amount were markedly reduced compared with the NC group (stool weight: 225.5 ± 47.11 mg vs. 20.75 ± 6.25 mg; stool amount: 238.4 ± 47.11 pellets vs. 22.6 ± 8.19 pellets; *p* < 0.05), indicating that stool weight and amount were important manifestations of constipation. CBLs and HBLs both prevented these changes, with higher doses producing greater effects. Notably, high-dose HBLs increased stool weight to 263.84 ± 66.70 mg and stool amount to 250.20 ± 66.88, which were approximately 12.7 times and 11.1 times higher than those in the MC group, respectively, representing a marked recovery towards NC levels (Figure 1b,c). Overall, HBLs showed more pronounced effects than CBLs on these parameters.

Gastrointestinal transit function can be assessed by measuring the time it takes for black stool to appear. Loperamide significantly prolonged this interval in the MC group compared with the NC group (226.4 ± 9.20 min vs. 146.4 ± 28.75 min; *p* < 0.05). In contrast, the PC group and both CBL and HBL treatment groups shortened the time to first black stool in a dose-dependent manner. High-dose CBLs and HBLs reduced this interval to 196.4 ± 18.40 min and 157.2 ± 2.64 min, respectively, representing a reduction of approximately 13.3% and 30.6% relative to the MC group (*p* < 0.05), with HBLs exerting the most pronounced effect.

Among all groups, the NC group exhibited the highest intestinal propulsion rate (1.05 ± 0.018%), followed by the PC group (0.855 ± 0.140%). Loperamide reduced the transit rate to 0.52 ± 0.025% in the MC group, whereas both CBLs and HBLs significantly increased this parameter (*p* < 0.05 vs. MC). High-dose HBLs increased the intestinal transit rate to 0.71 ± 0.072%, which was about 1.4 times higher than that of the MC group, indicating a partial restoration of gastrointestinal motility.

### 3.3. Effects of CBLs and HBLs on Serum Parameters

**Figure 2:**
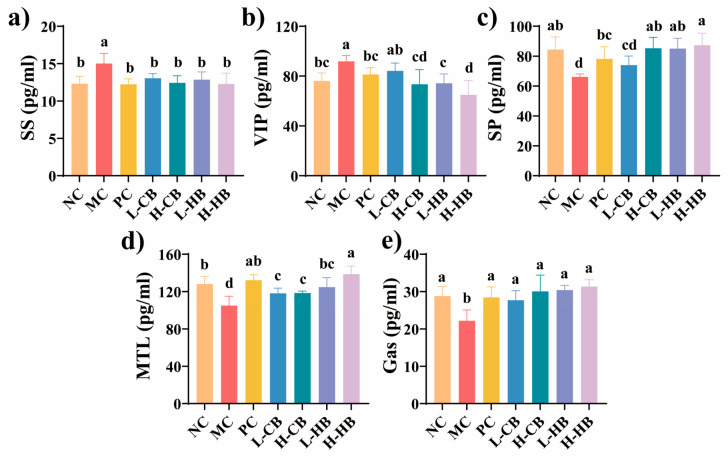
The effects of CBLs and HBLs on serum neurotransmitter level in constipated mice. Serum concentrations of (**a**) somatostatin (SS), (**b**) vasoactive intestinal peptide (VIP), (**c**) substance P (SP), (**d**) motilin (MTL), and (**e**) gastrin (Gas) were measured. Results are expressed as mean ± SD (*n* = 6). Different small letters indicate that values are significantly different at the *p* < 0.05 level.

As illustrated in Figure 2, the serum excitatory neurotransmitter (MTL, SP, and Gas) levels in the MC group significantly decreased (*p* < 0.05), while the inhibitory neurotransmitter (SS and VIP) levels increased (*p* < 0.05). These trends were consistent with previous reports describing loperamide-induced alterations in excitatory and inhibitory neurotransmitters related to gut motility [20,21]. Notably, therapeutic interventions with naloxone hydrochloride (PC group), HBLs, and high-dose CBLs significantly decreased the SS and VIP levels and substantially increased the levels of MTL, SP, and Gas compared with the MC group (*p* < 0.05), shifting these indicators toward the levels observed in the NC group. These findings suggested that both CBLs and HBLs exerted regulatory effects on hormones and neurotransmitters associated with gut motility, potentially helping prevent constipation.

### 3.4. Effects of CBLs and HBLs on Colon Gene Expressions

**Figure 3:**
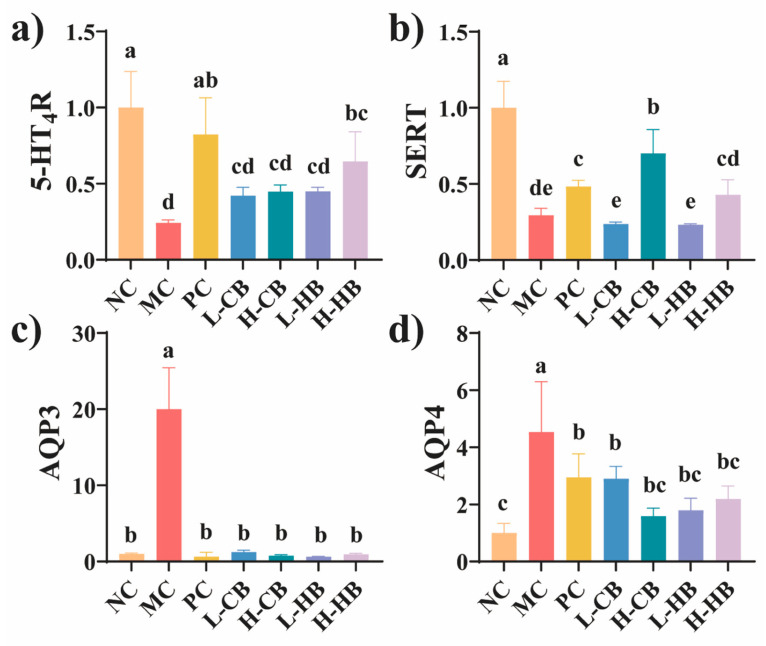
The effects of CBLs and HBLs on mRNA expression in colon tissues after induction of constipation. (**a**) mRNA expression of *5-HT*_4_*R*; (**b**) mRNA expression of *SERT*; (**c**) mRNA expression of *AQP3*; (**d**) mRNA expression of *AQP4*. Results are expressed as mean ± SD (*n* = 6). Different small letters indicate that values are significantly different at the *p* < 0.05 level.

Figure 3 presents the gene expression in the colon. Compared to the NC group, the MC group showed decreased expression of *5-HT*_4_*R* and *SERT* in the colon, while the expression of *AQP3* and *AQP4* increased (*p* < 0.05). In addition, with CBL treatment, colon expression of *AQP3* and *AQP4* significantly decreased (*p* < 0.05). Interestingly, high-dose HBL treatment led to a significant increase in colonic *5-HT*_4_*R* expression (*p* < 0.05), although low-dose treatment did not show a significant difference. However, high-dose CBL treatment resulted in a significant increase in colonic *SERT* expression (*p* < 0.05), with no significant difference observed for the low dose.

### 3.5. Effects of CBLs and HBLs on Colon Histological Morphology

**Figure 4:**
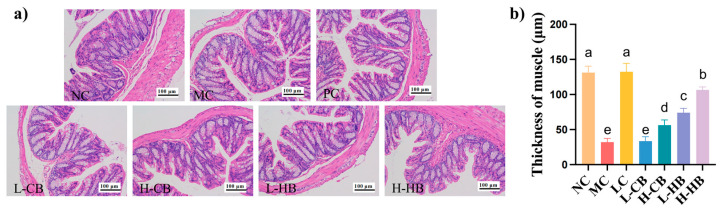
Protective effects of barley leaves on colonic histology in loperamide-induced constipation in mice. (**a**) Representative images of H&E staining in the colon tissues; (**b**) quantification of colonic muscular layer thickness. Results are expressed as mean ± SD (*n* = 3). Different small letters indicate that values are significantly different at the *p* < 0.05 level.

As shown in Figure 4, histopathological differences in colon tissues were observed among the groups. Representative H&E-stained images are shown in Figure 4a; the MC group had a reduction in the colonic muscular layer and structural disruption of the mucosal layer compared with the NC group. However, both the CBLs and HBLs groups showed protection against colon damage and preservation of intestinal barrier integrity. Interestingly, the H-HB group exhibited colon morphology similar to that of the NC group. Consistently, quantitative analysis of muscular layer thickness (Figure 4b) showed that loperamide significantly reduced this parameter (NC: 131.17 ± 8.31 μm vs. MC: 31.92 ± 4.90 μm, *p* < 0.05), while CBLs and HBLs partially restored it, with the most pronounced improvement in the H-HB group (106.56 ± 3.78 μm, *p* < 0.05 vs. MC). These observations suggested that BLs had a protective effect against constipation-induced colonic injury, with the most pronounced effect found in the H-HB group.

### 3.6. Effects of CBLs and HBLs on the Diversity of Fecal Microbiota

**Figure 5:**
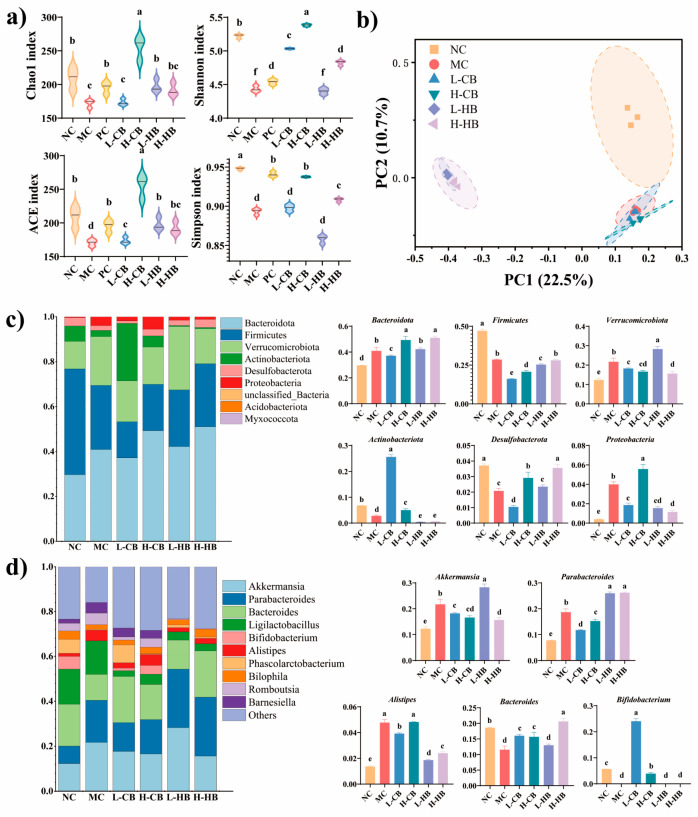
Effects of CBL and HBL treatment on the gut microbiota in constipated mice. (**a**) Alpha diversity (Chao1, ACE, Shannon, and Simpson indexes); (**b**) beta diversity determined using the PCoA method; (**c**) top ten gut flora microbial taxa at the phylum level; (**d**) top ten gut flora microbial taxa at the genus level. Results are expressed as mean ± SD (*n* = 3). Different small letters indicate that values are significantly different at the *p* < 0.05 level.

Comparative analysis revealed statistically significant differences in alpha-diversity indices (Chao1, ACE, Shannon, and Simpson; *p* < 0.05) between NC and MC groups (Figure 5a). Notably, low-dose CBLs failed to prevent the reduction in Chao1 and Simpson indices, whereas high-dose CBLs exhibited a significant protective effect on all four alpha diversity indices. Notably, unlike CBLs, high-dose HBLs markedly increased the Shannon and Simpson diversity indices (*p* < 0.05), with no significant changes observed in Chao1 or ACE indices (*p* > 0.05). Significant differences in beta diversity were detected between the MC and NC groups (*p* < 0.05), reflecting the impact of constipation on gut microbiota composition (Figure 5b). Although HBLs were administered, the microbial profiles remained unevenly distributed and the original structure of the fecal microbiome was not entirely reestablished. Intriguingly, in contrast to CBL treatments, HBLs intervention was differed from the microbiota of the MC group.

At the phylum level (Figure 5c), elevated abundances of *Bacteroidota*, *Actinobacteriota*, *Verrucomicrobiota*, and *Proteobacteria*, along with reduced levels of *Firmicutes* and *Desulfobacterota*, were observed in the MC group compared to the NC group. An overgrowth of *Verrucomicrobiota* might contribute to reduced levels of SCFAs [20]. However, both CBLs and HBLs (H-CB and H-HB groups) consistently suppressed *Verrucomicrobiota* levels while enhancing the abundance of *Desulfobacterota*. Notably, CBLs (H-B) uniquely restored *Actinobacteriota* levels to those observed in the NC group, whereas HBLs (H-HB) showed superior therapeutic efficacy compared to the H-CB group in reducing *Proteobacteria* abundance and promoting *Desulfobacterota* recovery.

At the genus level (Figure 5d), compared to the NC group, the MC group elevated abundances of *Akkermansia*, *Parabacteroides*, and *Alistipes*, along with reduced levels of *Bacteroides* and *Bifidobacterium*. CBLs markedly upregulated the abundance of *Bifidobacterium* and downregulated the abundance of *Akkermansia* and *Parabacteroides* (*p* < 0.05). In contrast to CBLs, HBLs were associated with a reduction in *Alistipes* abundance, with the high-dose intervention additionally suppressing *Akkermansia* levels. These findings highlight distinct modulation patterns between the two interventions, suggesting differential mechanisms on gut microbial restructuring.

**Figure 6:**
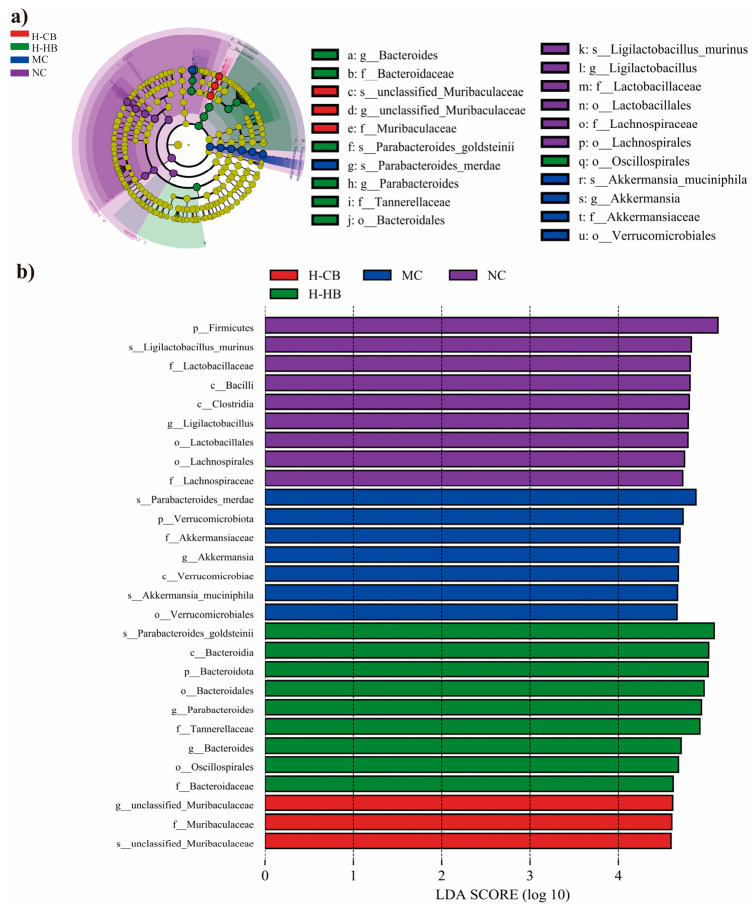
LEfSe analysis of intestinal microbiota among different mice groups. (**a**) Taxonomic cladogram obtained from LEfSe analysis. The brightness of each dot is proportional to its effect size. (**b**) LEfSe identified the most differentially abundant bacterial taxons among groups. Only taxa meeting an LDA significant threshold > 4 were shown (*n* = 3).

Focusing on specific bacterial taxa, distinct compositional modifications were observed following administration of loperamide, CBLs, and HBLs. Microbial community profiling using LEfSe methodology identified 21 operational taxonomic units (OUTs) exhibiting significant variation (Figure 6). *Verrucomicrobiota* at the genus level and *Parabacteroides_merdae* at the species level showed a significant response to the MC group mice. Altered gut microbiota could potentially contribute to the etiology of loperamide-induced constipation. The MC group was characterized by enrichment of Verrucomicrobiota-related taxa, including the genus *Akkermansia* and its species *Akkermansia muciniphila*, as well as the species *Parabacteroides_merdae*. In contrast, the H-HB group showed increased abundance of *Bacteroidota/Bacteroidia/Bacteroidales* and enrichment of the genera *Bacteroides* and *Parabacteroides*, with *Parabacteroides_goldsteinii* highlighted at the species level. The H-CB group was characterized by enrichment of *Muribaculaceae*, represented by an *unclassified_genus* within *Muribaculaceae* and an unclassified species within *Muribaculaceae*. The NC group exhibited enrichment of Firmicutes-related taxa and *Ligilactobacillus*, with *Ligilactobacillus_murinus* identified as a representative biomarker. Together, these species-level signatures provide additional resolution for understanding BL-associated microbial shifts in the constipation model.

### 3.7. Effects of CBLs and HBLs on the Production of SCFAs

**Figure 7:**
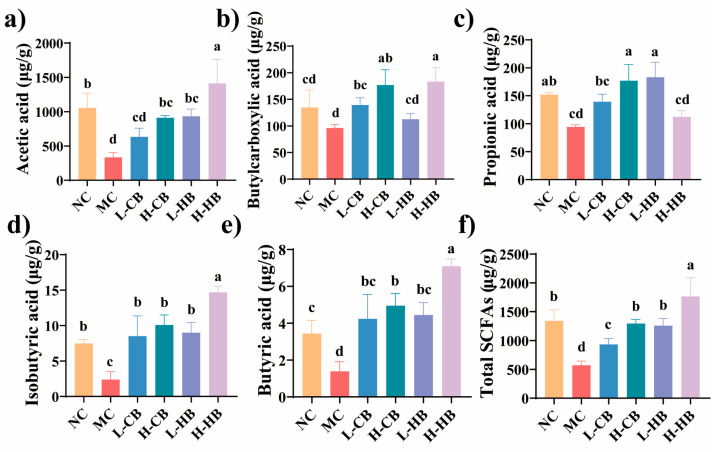
Effects of CBL and HBL treatments on SCFAs in stool contents (μg g^−1^). (**a**) Acetic acid; (**b**) butylcarboxylic acid; (**c**) propionic acid; (**d**) isobutyric acid; (**e**) butyric acid; (**f**) total SCFAs. Results are expressed as mean ± SD (*n* = 3). SCFA concentrations are reported as μg/g dry feces. Different small letters indicate that values are significantly different at the *p* < 0.05 level.

As illustrated in Figure 7, the amount of SCFAs in the MC group dramatically decreased (*p* < 0.05). Among SCFAs, acetic, butyric, and propionic acids were predominantly synthesized by gut microbes and made up more than 90% of the total. High-dose HBLs significantly elevated the levels of acetic, butylcarboxylic, isobutyric, and butyric acid compared with the MC group (*p* < 0.05). However, high-dose CBLs administration effectively enhanced the levels of SCFAs (*p* < 0.05).

### 3.8. Relationship Between SCFAs and Microbiota

**Figure 8:**
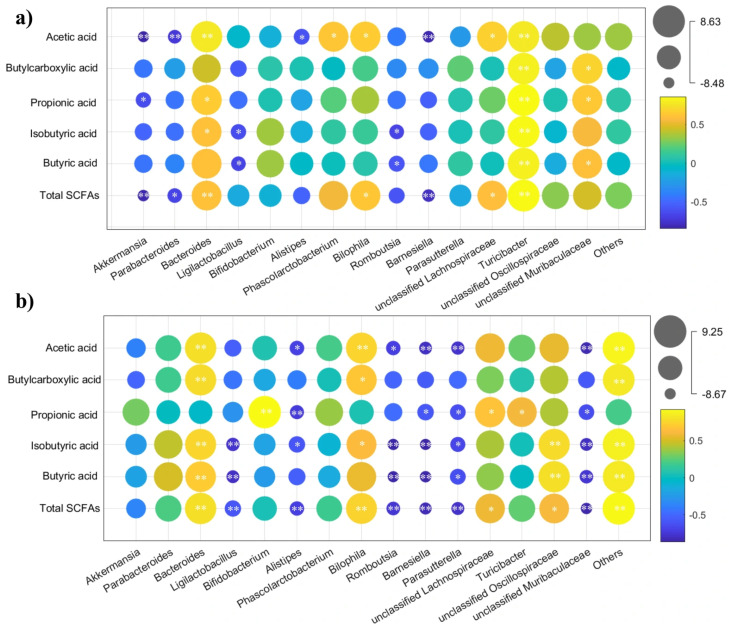
Heatmap of Spearman’s correlation coefficients between SCFAs and microbiota at genus level. (**a**) CBL treatment; (**b**) HBL treatment. The colors range from yellow (positive correlation) to blue (negative correlation), and significant correlations are marked with * *p* < 0.05, ** *p* < 0.01.

To investigate whether the prevention effects of CBLs and HBLs against constipation were mediated through alterations in gut microbiota, Spearman’s correlation was used to evaluate the connections between shifts in gut microbes and SCFAs. Following CBLs intervention, a strong positive association between *Bacteroides* and SCFA levels reached statistical significance (*p* < 0.05), while *Akkermansia* and *Parabacteroides* exhibited inverse correlations with SCFAs (Figure 8a, *p* < 0.05). However, in the HBL treatment group, *Bacteroides* maintained a positive correlation with SCFAs, and notable negative associations were observed for *Ligilactobacillus* and *Alistipes* (Figure 8b, *p* < 0.05).

## 4. Discussion

Constipation, resulting from a disruption in normal bowel function due to lifestyle factors such as low dietary fiber intake, inadequate hydration, or physical inactivity, is associated with various uncomfortable symptoms and represents a significant global public health challenge [21]. Dietary interventions are increasingly recognized as effective strategies for preventing constipation [22]. BLs have demonstrated various biological activities, including antiulcer, antioxidant, hypolipidemic, antidepressant, and antidiabetic potential [19]. However, their laxative effects and underlying mechanisms remain largely unexplored. Accordingly, two varieties of BLs (CBLs and HBLs) were selected, and their effects on constipation prevention and the underlying mechanisms were systematically investigated. Collectively, 7-day BLs administration effectively prevented loperamide-induced constipation, as evidenced by increased stool amount and weight, improved small intestinal motility, and shortened time to the first black stool. These protective outcomes were accompanied by coordinated changes in gut motility-related neurotransmitters, serotonin-related gene expression, aquaporins, gut microbiota, and SCFAs.

The regulation of neurotransmitters related to gut motility was observed following BL treatment. Evidence from previous studies has highlighted the involvement of enteric nervous system dysfunction in the development of constipation [23]. SS and VIP are inhibitory peptide neurotransmitters, while MTL, SP, and Gas are excitatory peptide neurotransmitters. MTL influences water and electrolyte transport, promotes gastrointestinal peristalsis, and stimulate secretions of hydrochloric acid secretion, pancreatic juices, and bile [24]. SP induces phasic contractions in gastrointestinal smooth muscles via neurokinin receptor activation. Gas is synthesized and secreted by G cells and promotes gastrointestinal motility through RAS-MAP kinase pathway activation [25,26]. Conversely, VIP suppresses gastrointestinal smooth muscle tone, thereby reducing intestinal motility [27]. Similarly, SS binds to its receptors on smooth muscle tissue and inhibits acetylcholine release, ultimately leading to decreased gastrointestinal peristaltic activity [28]. In our study, loperamide decreased serum MTL, SP, and Gas and increased SS and VIP, whereas CBL and HBL supplementation partially reversed these alterations, shifting the overall profile toward the NC group. These findings aligned with previous reports that loperamide disrupts neurotransmitters and impairs gut motility [20,21].

Gut motility enhances 5-HT by activating *5-HT*_4_*R* receptors, which stimulate neurotransmitter release and promote intestinal contractions [29]. *SERT* transports 5-HT back into cells after activation, and its levels rise in response to increased 5-HT to prevent overstimulation [30]. Our gene expression data showed that high-dose CBLs markedly increased *SERT* expression, whereas high-dose HBLs increased *5-HT*_4_*R* with minimal effect on *SERT*. These patterns suggest that CBLs may primarily affect 5-HT clearance/homeostatic regulation, while HBLs may preferentially influence 5-HT_4_R-related signaling. Overall, BLs may modulate serotonin-related pathways. However, the functional effects of BL-induced changes in *SERT* and *5-HT*_4_*R* and the direct contribution of *SERT/5-HT*_4_*R* to gut motility still need further verification.

Clone aquaporins, particularly *AQP3* and *AQP4*, play crucial roles in water transport and are potential targets for constipation prevention [31,32]. *AQP3* is predominantly found in colonic epithelial cells and is involved in water reabsorption. *AQP4* is widely distributed in the colon and gastric fundus and contributes to water and mucus secretion [31]. Compared with the MC group, the NC group exhibited notably higher levels of *AQP3* and *AQP4*. Conversely, BLs attenuated these increases. These findings suggest that BLs may help modulate colonic water transport and thereby contribute to improved fecal hydration.

Gut microbiota dysbiosis disrupts normal bowel movements and intestinal peristalsis in constipation models [22]. BLs exhibited regulatory effects on the gut microbiota of constipated mice, partially restoring microbial diversity and community structure. MC group increased the abundances of *Akkermansia*, *Parabacteroides*, and *Alistipes* but decreased the abundances of *Bacteroides*. Interestingly, both CBLs and HBLs prevented the constipation-associated shifts by increasing the abundance of *Bacteroides* and reducing that of *Akkermansia*. Moreover, CLBs specifically decreased the abundance of *Parabacteroides*, whereas HLBs significantly lowered the abundance of *Alistipes*. *Bacteroides* is a dominant group known for producing acetate, propionate, and succinate and has been reported to decrease in constipated mice [27]. *Akkermansia*, a mucin-degrading bacterium, may disrupt mucin degradation processes when excessively enriched, leading to impaired intestinal barrier function [33]. Wang et al. reported an increase abundance of *Akkermansia* increased in constipated mice [34]. Previous studies have shown that *Alistipes* is associated with the development of intestinal injury and colorectal cancer [35]. Given the small sample size for microbiota sequencing (*n* = 3 pooled samples per group), these taxonomic findings should be interpreted cautiously and are considered exploratory and supportive. Nevertheless, the direction of microbial changes was consistent with improvements in fecal parameters, intestinal transit, and SCFA profiles.

SCFAs are microbial metabolites generated in the colon and are known to exert various biological effects on gut homeostasis and immune regulation [36]. SCFAs can elevate intestinal osmotic pressure and stimulate intestinal peristalsis [37]. We found that high dose of BLs (both CBLs and HBLs) could enhance the levels of SCFAs. This increase may be related to the enrichment of SCFA-producing taxa such as *Bacteroides*. Increased levels of SCFAs may directly or indirectly enhance *5-HT* secretion, thereby promoting intestinal motility in constipated mice [38]. Furthermore, *Ligilactobacillus* has been reported to promotes 5-HT transport by increasing *SERT* expression in intestinal cells [39]. CBLs markedly upregulated the abundance of *Bifidobacterium*, which may have partially explained the distinct pattern of serotonin-related gene modulation observed in the CBL-treated groups. Correlation analysis further suggested association between *Bacteroides* and fecal SCFAs levels following treatment with BLs, whereas *Akkermansia* and *Parabacteroides* were negatively correlated with SCFAs in the CBL group, and *Ligilactobacillus* and *Alistipes* showed negative links in the HBLs group. Although multiple outcomes showed consistent improvements, direct causal links among microbial shifts, SCFA alterations, and neurotransmitter/serotonin-related signaling were not experimentally confirmed.

Potential biochemical differences between CBLs and HBLs may explain their distinct physiological effects. HBLs showed a higher content of dietary fiber and protein, whereas CBLs exhibited higher contents of total phenolics and flavonoids. The higher dietary fiber content in HBLs may provide more fermentable substrates for SCFA production, whereas the higher phenolic and flavonoid contents of CBLs may contribute to differential modulation of specific bacterial taxa and serotonin-related gene expression. Moreover, while BLs showed constipation-preventive effects, the key active ingredients and their specific roles were not elucidated. Future studies are needed to fully understand the causal relationships among microbial shifts, SCFA alterations, and neurotransmitter/serotonin-related signaling. Nevertheless, this study provides preliminary evidence for the preventive potential of different BLs against loperamide-induced constipation. This work lays a foundation for further mechanistic studies and dietary applications.

## 5. Conclusions

**Figure 9:**
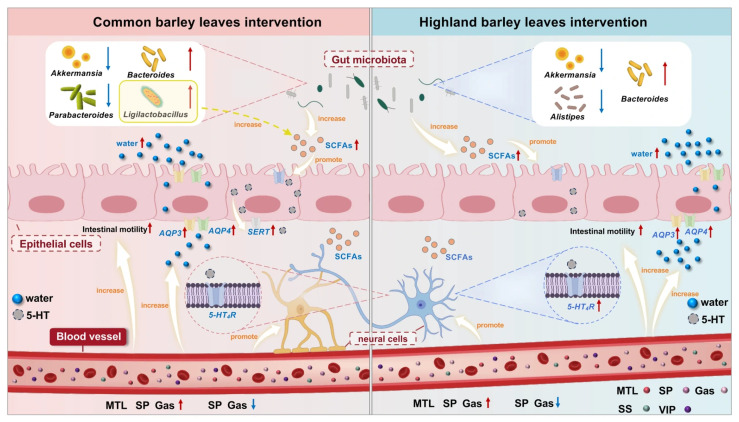
A summary of possible mechanisms of CBLs and HBLs in preventing constipation. Red arrows indicate increases and blue arrows indicate decreases.

In summary, this study demonstrated that BLs exerted significant constipation-preventive effects in a loperamide-induced mice model, highlighting their potential for intestinal health regulation. The findings indicated that CBLs and HBLs had significant potential in preventing constipation by improving physiological parameters; increasing serum levels of Gas, SP and MTL; decreasing serum levels of SS and VIP; restoring colon *AQP3*, *AQP4*, and *5-HT*_4_*R* mRNA expression; improving fecal SCFAs content; and modulating gut microbiota composition by increasing *Bacteroides* abundance and reducing that of Akkermansia (Figure 9). Histopathological analyses further supported these findings. CBLs and HBLs also exhibited distinct mechanisms. High-dose CBLs significantly increased *SERT* expression, whereas HBLs significantly decreased *Alistipes* abundance and increased SCFA levels. Although this study provides evidence for BLs as promising functional food ingredients for constipation prevention, future research still needs to conduct in-depth exploration in multiple aspects to comprehensively evaluate its safety and efficacy and reveal the specific mechanism of its treatment for constipation prevention.

## Figures and Tables

**Table 1:** The sequence of primers used to determine gene expression in colon tissue.

	Primer Sequence	
	Forward	Reverse
GAPDH	TCCTGCACCACCAACTGCT	GTCAGATCCACGACGGACACA
5-HT_4_R	AGTTCCAACGAGGGTTTCAGG	CAGCAGGTTGCCCAAGATG
AQP4	TGATTCCAAACGAACTGATGTT	ATAACTGCGGGTCCAAAAGATT
AQP3	GCCAAGGTAGGATAGCAAATAA	TTGAAAACTTGGTCCCTTGC
SERT	TGGCAGGATCACATTACAGGG	TCGCTCCTCGAAAATGGAGAT

**Table 2:** Chemical composition of CBLs and HBLs.

	Content (mg per g Dry Weight)	
	CLBs	HLBs
Dietary fiber	448.4 ± 2.1 ^b^	475.7 ± 1.7 ^a^
Protein	219.0 ± 3.4 ^b^	282.4 ± 1.1 ^a^
Total phenolics	21.4 ± 0.2 ^a^	7.42 ± 0.1 ^b^
Total flavonoids	20.69 ± 0.46 ^a^	1.01 ± 0.06 ^b^

Results were expressed as mean ± SD (*n* = 3). Different small letters indicate that values are significantly different at the *p* < 0.05 level.

## Data Availability

The original contributions presented in this study are included in the article. Further inquiries can be directed to the corresponding author.

## Abbreviations

BL	Barley leaf
CBL	Common barley leaf
HBL	Hulless barley leaf
GI	Gastrointestinal
SCFAs	Short-chain fatty acids
SP	Substance P
MTL	Motilin
Gas	Gastrin
SS	Somatostatin
VIP	Intestinal peptide
PCoA	Principal coordinate analysis
LEfSe	Linear discriminant analysis effect size
